# Hard, harder, hardest: principal stratification, statistical identifiability, and the inherent difficulty of finding surrogate endpoints

**DOI:** 10.1186/1742-7622-11-14

**Published:** 2014-08-26

**Authors:** Julian Wolfson, Lisa Henn

**Affiliations:** 1 University of Minnesota Division of Biostatistics, A460 Mayo Building MMC 303, 420 Delaware St SE, Minneapolis MN, USA

**Keywords:** Surrogate endpoint, Principal stratification, Causal inference, Statistical identifiability

## Abstract

In many areas of clinical investigation there is great interest in identifying and validating surrogate endpoints, biomarkers that can be measured a relatively short time after a treatment has been administered and that can reliably predict the effect of treatment on the clinical outcome of interest. However, despite dramatic advances in the ability to measure biomarkers, the recent history of clinical research is littered with failed surrogates. In this paper, we present a statistical perspective on why identifying surrogate endpoints is so difficult. We view the problem from the framework of causal inference, with a particular focus on the technique of principal stratification (PS), an approach which is appealing because the resulting estimands are not biased by unmeasured confounding. In many settings, PS estimands are not statistically identifiable and their degree of non-identifiability can be thought of as representing the statistical difficulty of assessing the surrogate value of a biomarker. In this work, we examine the identifiability issue and present key simplifying assumptions and enhanced study designs that enable the partial or full identification of PS estimands. We also present example situations where these assumptions and designs may or may not be feasible, providing insight into the problem characteristics which make the statistical evaluation of surrogate endpoints so challenging.

## Introduction

### Background

Randomized clinical trials are well-suited to answering the question of whether a particular treatment affects an outcome. Randomization ensures that on average, all covariates–whether measurable or unmeasurable, known or unknown–are equally distributed between treatment groups. Differences in outcomes between these groups can thus be attributed to the treatment alone, and comparisons of randomized treatment groups yield what are rightly termed causal effects. But clinical trials are often lengthy and costly, particularly when the outcome of interest is relatively rare (e.g., occurrence of myocardial infarction, infection with HIV). Long trials provide high-quality evidence about the efficacy of treatments, but they can delay research progress as scientists must wait for results to learn how treatments might be improved. As a result, in many areas of clinical investigation there is great interest in identifying surrogate endpoints, outcomes (often biomarkers) that can be measured a relatively short time after a treatment has been administered and which reliably predict the effects of treatment on clinical outcomes of interest.

A validated surrogate permits a future treatment to be evaluated much more quickly, since observed effects on the surrogate can be translated into an expected level of clinical efficacy without the need to carry the study forward and record clinical outcomes. A valid surrogate can also give insight into the biological mechanisms of treatment. However, declaring a biomarker to be a surrogate when it is merely correlated with clinical risk can divert valuable resources toward scientific dead ends. Treatments targeting candidate surrogate biomarkers have often been ineffective, with failures ranging across diseases including cardiovascular disease, cancer, and osteoporosis [1].

Because a candidate surrogate is measured some time after treatment has been administered, its levels may be affected by treatment, thereby introducing possible confounding of the association between the surrogate and the outcome – see, e.g., Figure one of Wolfson and Gilbert [2]. This confounding, also termed selection bias, can invalidate traditional analyses which estimate the effect of treatment on the outcome conditional on the level of the candidate surrogate. It is particularly difficult to control for this confounding by adjusting for baseline covariates since candidate surrogate biomarkers often are themselves poorly understood and the relevant adjustment variables are unclear. The possible influence of unmeasured confounders within individual studies can be mitigated by meta-analytic techniques [3,4], but the surrogate endpoint problem often will be of interest in situations where it is too costly or too time-consuming to perform multiple trials measuring both the biomarker and the clinical outcome. For this paper, we therefore restrict attention to approaches for assessing surrogate value using data from a single trial.

The potential for unmeasured confounding in the surrogate endpoint problem has motivated the development and application of statistical methods for causal inference in this setting. These methods propose estimands based on counterfactuals–also called potential outcomes–whose basic theory is described elsewhere [5,6]. The key characteristic of these causal estimands is that they quantify within-person treatment effects, which are by definition free from confounding.

Joffe and Greene [7] identified four approaches for evaluation of surrogate outcomes and grouped them into two paradigms. The *causal effects* paradigm considers individual treatment effects when the candidate surrogate is fixed at different values. Prentice [8] described a set of criteria for validating a surrogate using observed data from a single randomized trial; the criteria are closely related to those proposed by Baron and Kenny [9] to assess mediation, and have been widely applied (e.g., [10-12]). More recent work [13-16] has focused on quantifying counterfactual *direct* and *indirect effects* of treatment, and though most of this work is framed in terms of estimating mediating effects, it is also applicable to surrogate endpoint assessment as strong mediators are likely to be good surrogates although the converse need not be true [17]. However, some authors [18] have criticized this approach to evaluating surrogate endpoints on the basis that it posits a hypothetical manipulation of the biomarker value which may be implausible.

The second paradigm described by Joffe and Greene is the *causal association* paradigm, which considers the association between the effect of treatment on the surrogate and the effect of treatment on the outcome. The main strategy under this paradigm is principal stratification, which was proposed by Frangakis and Rubin [18] and has been developed further in a number of recent papers [2,19-22]. Evaluating surrogacy via principal stratification relies on partioning subjects according to the (counterfactual) biomarker values that would have been observed under assignment to the control and treatment arms. Since the resulting *principal strata* are assumed to be independent of treatment (i.e., they are baseline subject characteristics), treatment remains randomized and hence causal effects can be estimated through calculation of a contrast in outcomes between those assigned to treatment and to control within each stratum. The final assessment of surrogate value involves quantifying the causal effects of treatment within defined sets of principal strata. Because each participant is assigned either to the control or to the treatment arm, only one of the two potential biomarker values in the pair defining the stratum is known; the other is counterfactual. Principal stratification estimands are therefore not usually statistically identifiable unless strong, untestable assumptions are made.

Previous work has focused on describing assumptions and developing PS-based approaches in specific scenarios [2,19]. This paper takes a broader view by characterizing the key scientific, statistical and study design aspects that challenge the identification of principal stratification estimands for quantifying the surrogate value of a biomarker. In each specific scenario, the degree of statistical nonidentifiability of PS estimands can be viewed as a rough measure of the inherent difficult of the statistical evaluation of surrogate endpoints in that scenario. Exploring the identifiability of PS estimands can therefore explain why medical researchers have been relatively successful in identifying surrogates in some contexts but not others. And, perhaps more importantly, understanding the aspects of a scientific problem which help or hinder the statistical assessment of surrogate value can anticipate the degree to which novel study designs and statistical analyses will be helpful in the search for surrogate endpoints. When the key assumptions described below are plausible, causal PS analyses may be vital for identifying promising surrogates and it is advisable to conduct large endpoint-driven studies which collect the biomarker data necessary to enable these analyses. In settings where many of the assumptions are violated, causal PS analyses may have relatively little to offer and researchers should pursue other approaches–such as additional laboratory experiments and mechanistic studies–to gain insight about potential surrogate biomarkers.

In the Section “Principal stratification for assessing surrogate value” describes three common simplifying assumptions the plausibility of which can significantly influence the statistical identifiability of principal stratification estimands. The Section “Auxiliary data and augmented study designs” outlines how auxiliary data and novel study designs can be used to identify estimands of interest. In the Section “Example scenarios”, we present four example scenarios that illustrate how the characteristics of the disease process, study population, and trial design can combine to make the statistical assessment of surrogate endpoints relatively straightforward or extremely difficult. We conclude with a brief discussion in Section “Conclusion”.

## Principal stratification for assessing surrogate value

### Notation and setup

Consider a randomized trial where subjects *i*=1,…,*n* randomly are assigned at baseline to one of two treatments (*Z*_*i*_=0,1) and followed for a binary outcome *Y*_*i*_. A biomarker, *S*_*i*_, is measured at some time *τ*>0, and we assume that *τ* is the same for all subjects. Biomarkers might include values derived from a lab-based assay or a patient’s health status at *τ*. In some situations, due to the occurrence of the clinical outcome *Y* prior to *τ* or due to other reasons, it may not be possible to measure *S*_*i*_ at *τ*. For example, in HIV vaccine trials where *Y*_*i*_ indicates infection with HIV and *S*_*i*_ is some immune response to the vaccine, infection with HIV prior to *τ* precludes the meaningful measurement of *S*_*i*_ at *τ*. We let $A_{i}^{\tau }$ denote whether the biomarker is observable at *τ*. When $A_{i}^{\tau }=0$, *S*_*i*_ is undefined, which we denote *S*_*i*_=⋆. Note that *S*_*i*_ may be unobserved but not undefined in cases of drop-out or loss-to-follow-up; we do not discuss the complexities of handling missing but well-defined biomarker values in this paper. Lastly, we assume that a vector of baseline covariates *W*_*i*_ is available for each subject.

### Principal stratification

In our work, we let $\left(A_{\text{iz}}^{\tau },S_{\text{iz}},Y_{\text{iz}}\right)$ be the counterfactual values of $\left(A_{i}^{\tau },S_{i},Y_{i}\right)$ under treatment assignment *Z*_*i*_=*z* for each study participant *i*. For simplicity of presentation, we generally suppress the subscript *i*. Note the distinction between these counterfactuals, which describe potential outcomes under different settings of *Z*, and those employed by, e.g., Robins and Greenland [23], which refer to potential outcomes when both *Z* and *S* are set to certain values.

For assessing surrogate value in the context of a randomized trial, Frangakis and Rubin [18] suggested comparing the treatment effect on the outcome of interest within two classes of *principal strata*:

$$G_{1}:\left\{\left\{S_{0},S_{1}\right\}:S_{0}=S_{1}\right\}\;\;\text{and}\;\;G_{2}:\left\{\left\{S_{0},S_{1}\right\}:S_{0}\neq S_{1}\right\}$$

Within these strata–which are not affected by treatment and hence play the same role as baseline covariate–treatment assignments remain randomized and hence it is trivial to estimate the causal effect of treatment. For a good surrogate, we hope to see a small or no treatment effect within principal strata from $G_{1}$, i.e., those in which subjects experience no causal treatment effect on *S*, and some treatment effect on the outcome within principal strata from $G_{2}$, i.e., those in which there is some treatment effect on *S*.

Gilbert and Hudgens [19] extended on Frangakis and Rubin’s work, and proposed to assess surrogate value based on contrasts of the estimands

(1) $$\begin{array}{lcr} R_{0}\;\left(S_{0},S_{1}\right) & \equiv & P\;\left(Y_{0}=1\;|\;S_{0},S_{1},A_{0}^{\tau }=A_{1}^{\tau }=1\right)\;\;\text{and} \end{array}$$

(2) $$\begin{array}{lcr} R_{1}\;\left(S_{0},S_{1}\right) & \equiv & P\;\left(Y_{1}=1\;|\;S_{0},S_{1},A_{0}^{\tau }=A_{1}^{\tau }=1\right). \end{array}$$

The conditioning event $\left\{A_{0}^{\tau }=A_{1}^{\tau }=1\right\}$ is necessary to ensure that the joint values of (*S*_0_,*S*_1_) are well defined. Based on these estimands, Gilbert and Hudgens defined a principal surrogate as a biomarker satisfying Average Causal Necessity and Average Causal Sufficiency:

**Average causal necessity:***R*_0_ (*S*_0_,*S*_1_)=*R*_1_ (*S*_0_,*S*_1_) for all *S*_1_=*S*_0_.

**Average causal sufficiency:** There exists a constant *C*≥0 such that *R*_1_ (*S*_0_,*S*_1_)≠*R*_0_ (*S*_0_,*S*_1_) for all |*S*_0_−*S*_1_|>*C*.

Wolfson and Gilbert [2] considered the identifiability and estimation of Equations 1 and (2) in the context of HIV vaccine trials. Here, we explore the identifiability of these estimands in a wider variety of contexts.

### Basic assumptions

In what follows, we describe several basic assumptions which are generally uncontroversial in the randomized trial setting. Without these assumptions, estimation would be virtually impossible; the remainder of this paper focuses on stronger assumptions that may be defensible in certain settings and that can help identify estimands for assessing surrogate value. The basic assumptions are:

Stable unit treatment value assumption (SUTVA):

1. **[No interference]** The potential outcomes $\left(Y_{0},Y_{1},A_{0}^{\tau },A_{1}^{\tau },S_{0},S_{1}\right)$ for one subject are independent of the treatment assignments of other subjects, i.e., there is no “interference” between experimental units.

2. **[Consistency]** For an individual receiving treatment *Z*=*z* and with observed outcome *Y*, we have *Y*=*Y*_*z*_, i.e., the observed outcome is equal to the potential outcome under the treatment actually received.

**Ignorable treatment assignments:***Z* is independent of $\left(Y_{0},Y_{1},A_{0}^{\tau },A_{1}^{\tau },S_{0},S_{1}\right)$.

The validity of the “No Interference” part of SUTVA may be questioned when the *Y* represents infection with a communicable disease, but it is defensible in these settings if a trial enrolls a small fraction of the at-risk population. Work by Hudgens and Halloran [24] discusses relaxation of SUTVA. Ignorable Treatment Assignments will generally hold in a randomized trial where blinding is maintained.

### Estimands and identifiability

In what follows, we study the risk estimands *R*_1_ and *R*_0_ under a variety of scenarios and assumptions. We focus on the concept of nonparametric identifiability, i.e., whether it is possible to obtain arbitrarily precise estimates of these quantities given an infinite sample size, making no further assumptions regarding the data distribution. If *S*_0_ and *S*_1_ respectively were to take on discrete values in {*s*_01_,*s*_02_,…,*s*_0*K*_} and {*s*_11_,*s*_12_,…,*s*_1*K*_}, then assuming a sufficiently large sample size, *R*_0_ and *R*_1_ would be nonparametrically identifiable if *R*_0_ (*s*_0*j*_,*s*_1*k*_) and *R*_1_ (*s*_0*j*_,*s*_1*k*_) could be estimated precisely from observed data for all *j* and *k*.

The nonparametric identifiability properties of *R*_1_ and *R*_0_ can be understood by applying Bayes Rule:

(3) $$\begin{array}{ll} R_{1}\left(s_{0},s_{1}\right) & \;=\;\frac{f\;\left(s_{0},s_{1}|Y_{1}\;=\;1,A_{0}^{\tau }\;=\;A_{1}^{\tau }\;=\;1\right)P\;\left(Y_{1}\;=\;1|A_{0}^{\tau }\;=\;A_{1}^{\tau }\;=\;1\right)}{f\;\left(s_{0},s_{1}|A_{0}^{\tau }\;=\;A_{1}^{\tau }\;=\;1\right)} \end{array}$$

(4) $$\begin{array}{ll} R_{0}\;\left(s_{0},s_{1}\right) & \;=\;\frac{f\;\left(s_{0},s_{1}|Y_{0}\;=\;1,A_{0}^{\tau }\;=\;A_{1}^{\tau }\;=\;1\right)P\;\left(Y_{0}\;=\;1|A_{0}^{\tau }\;=\;A_{1}^{\tau }\;=\;1\right)}{f\;\left(s_{0},s_{1}|A_{0}^{\tau }=A_{1}^{\tau }\;=\;1\right)} \end{array}$$

where *f* (*s*_0_,*s*_1_ | ·) and *f* (*s*_0_,*s*_1_) are joint densities (or probability mass functions) of (*S*_0_,*S*_1_). For simplicity, we assume that these densities or p.m.f.’s exist.

To evaluate Average Causal Necessity and Sufficiency, one must contrast the risks *R*_1_ (*s*_0_,*s*_1_) and *R*_0_ (*s*_0_,*s*_1_) for different values of (*s*_0_,*s*_1_). ACN and ACS above are stated in terms of the risk difference,

(5) $$\begin{array}{l} \text{RD}\;\left(s_{0},s_{1}\right)\\ \;=\;R_{1}\;\left(s_{0},s_{1}\right)-R_{0}\;\left(s_{0},s_{1}\right)\\ \;=\left[\;f\;\left(s_{0},s_{1}|Y_{1}\;=\;1,A_{0}^{\tau }\;=\;A_{1}^{\tau }\;=\;1\right)P\;\left(Y_{1}\;=\;1|A_{0}^{\tau }\;=\;A_{1}^{\tau }\;=\;1\right)\right)\\ \;\left(-\;f\;\left(s_{0},s_{1}|Y_{0}\;=\;1,A_{0}^{\tau }\;=\;A_{1}^{\tau }\;=\;1\right)P\;\left(Y_{0}\;=\;1|A_{0}^{\tau }\;=\;A_{1}^{\tau }\;=\;\;1\right)\;\right]/f\;\left(s_{0},s_{1}|A_{0}^{\tau }\;=\;A_{1}^{\tau }\;=\;1\;\right) \end{array}$$

They may also be stated in terms of the relative risk,

(6) $$\begin{array}{l} \text{RR}\;\left(s_{0},s_{1}\right)\;=\;\frac{R_{1}\;\left(s_{0},s_{1}\right)}{R_{0}\;\left(s_{0},s_{1}\right)}\\ \;=\frac{f\;\left(s_{0},s_{1}|Y_{1}\;=\;1,A_{0}^{\tau }\;=\;A_{1}^{\tau }\;=\;1\right)P\;\left(Y_{1}\;=\;1|A_{0}^{\tau }\;=\;A_{1}^{\tau }\;=\;1\;\right)}{f\;\left(s_{0},s_{1}|Y_{0}\;=\;1,A_{0}^{\tau }\;=\;A_{1}^{\tau }\;=\;1\right)P\;\left(Y_{0}\;=\;1|A_{0}^{\tau }\;=\;A_{1}^{\tau }\;=\;1\right)} \end{array}$$

where ACN holds if *RD* (*s*_0_,*s*_1_)=0≡*RR* (*s*_0_,*s*_1_)=1 for *s*_1_=*s*_0_ and ACS holds if *RD* (*s*_0_,*s*_1_)≠0≡*RR* (*s*_0_,*s*_1_)≠1 for *s*_1_≠*s*_0_.

In the most general case where no assumptions beyond the Basic Assumptions above are made, neither *R*_1_, *R*_0_, nor their contrasts *RD* and *RR* are statistically identifiable. This is clear since none of the terms on the right-hand sides of (3)-(6) is identifiable: Neither $\left(A_{1}^{\tau },A_{0}^{\tau }\right)$ nor (*S*_0_,*S*_1_) can be observed simultaneously on a subject, hence observed data do not reveal membership in the stratum defined by $A_{1}^{\tau }=A_{0}^{\tau }=1$ nor do they allow estimation of the joint distribution of *S*_0_ and *S*_1_. In the next section, we discuss assumptions that allow some or all of the expressions in (3)-(6) to be identified from observed data, and describe situations in which these assumptions may be plausible. This will lead naturally to Section “Example scenarios”, where we describe scenarios that vary according to the inherent difficulty of identifying principal surrogate estimands and hence evaluating the surrogate value of biomarkers.

## Simplifying assumptions

We begin with a fundamental simplifying assumption without which it is very difficult to achieve statistical identifiability of *R*_0_ and *R*_1_:

**[SA1] The biomarker** ***S* is defined on all subjects at time** ***τ*, i.e.,**$A^{\tau }=A_{0}^{\tau }=A_{1}^{\tau }=1$** for all subjects.**

[SA1] is likely to hold in situations where *S* can be measured shortly after treatment is administered at baseline, and (trivially) when *S* is an intermediate outcome, e.g., two-year progression-free survival with prostate cancer when the clinical outcome of interest is five-year overall survival.

If [SA1] holds, there is no need to condition on $\left(A_{0}^{\tau },A_{1}^{\tau }\right)$ and hence (3)-(6) simplify to

(7) $$\;\begin{array}{ll} R_{1}\;\left(s_{0},s_{1}\right) & \;=\frac{f\;\left(s_{0},s_{1}\;|\;Y_{1}=1\right)P\;\left(Y_{1}=1\right)}{f\;\left(s_{0},s_{1}\right)} \end{array}$$

(8) $$\;\begin{array}{ll} R_{0}\;\left(s_{0},s_{1}\right) & \;=\frac{f\;\left(s_{0},s_{1}\;|\;Y_{0}=1\right)P\;\left(Y_{0}=1\right)}{f\;\left(s_{0},s_{1}\right)} \end{array}$$

(9) $$\begin{array}{ll} \text{RD}\;\left(s_{0},s_{1}\right) & \;=\;\frac{f\;\;\left(s_{0},s_{1}|Y_{1}\;=\;1\right)P\;\left(Y_{1}\;=\;\;1\right)\;-\;f\;\left(s_{0},s_{1}|Y_{0}\;=\;\;1\right)P\;\left(Y_{0}\;=\;1\right)}{f\;\left(s_{0},s_{1}\right)} \end{array}$$

(10) $$\;\begin{array}{ll} \text{RR}\;\left(s_{0},s_{1}\right) & \;=\frac{f\;\left(s_{0},s_{1}\;|\;Y_{1}=1\right)P\;\left(Y_{1}=1\right)}{f\;\left(s_{0},s_{1}\;|\;Y_{0}=1\right)P\;\left(Y_{0}=1\right)} \end{array}$$

By the Basic Assumptions, *P* (*Y*_1_=1) and *P* (*Y*_0_=1) are identifiable and can be estimated as the sample mean of *Y* among subjects assigned to *Z*=1 and *Z*=0, respectively. The joint densities denoted by *f* remain non-identifiable, but the subgroups within which these densities are to be estimated (*Y*_1_=1 and *Y*_0_=1) are identified. While [SA1] must hold exactly to achieve simplifications of (7)-(10), if the proportion of subjects with *A*^*τ*^=0 is very small then it may be plausible to discard these subjects from the analysis and proceed under [SA1].

[SA1] represents a first “layer” of non-identifiability, below which lie additional identifiability challenges. Hence, for the remainder of this section the estimands we present implicitly condition on $A_{0}^{\tau }=A_{1}^{\tau }=1$.

**[SA2] Constant biomarker values under placebo** ***(S***_***0***_ ***=c)***

Sometimes, the task of imputing the joint biomarker values (*S*_0_,*S*_1_) is made simpler by placing restrictions on their joint distribution. These restrictions may reflect inherent features of the biomarkers themselves, or the manner in which treatment and biomarkers interact. One specific restriction that aids identifiability is based on the assumption that *S*_0_=*c* for all subjects, i.e., subjects receiving the placebo achieve the same (often null) biomarker value. This assumption may be plausible when the biomarker of interest directly quantifies response to treatment and has little natural variability absent that treatment, for example if *S* were the serum concentration of a particular drug metabolite which does not naturally occur in the body.

Under [SA2], the principal strata (*S*_0_,*S*_1_)=(*c*,*S*_1_) and thus are defined fully by *S*_1_. When [SA1] and [SA2] both hold, (7)-(10) further simplifies to

(11) $$\;\begin{array}{ll} R_{1}\;\left(s_{1}\right) & =\frac{f\;\left(s_{1}\;|\;Y_{1}=1\right)P\;\left(Y_{1}=1\right)}{f\;\left(s_{1}\right)} \end{array}$$

(12) $$\;\begin{array}{ll} R_{0}\;\left(s_{1}\right) & =\frac{f\;\left(s_{1}\;|\;Y_{0}=1\right)P\;\left(Y_{0}=1\right)}{f\;\left(s_{1}\right)} \end{array}$$

(13) $$\begin{array}{ll} \text{RD}\;\left(s_{1}\right) & \;=\;\frac{f\;\left(s_{1}|Y_{1}\;=\;1\right)P\;\left(Y_{1}\;=\;1\right)-f\;\left(s_{1}|Y_{0}\;=\;1\right)P\;\left(Y_{0}\;=\;1\right)}{f\;\left(s_{1}\right)} \end{array}$$

(14) $$\;\begin{array}{ll} \text{RR}\;\left(s_{1}\right) & =\frac{f\;\left(s_{1}\;|\;Y_{1}=1\right)P\;\left(Y_{1}=1\right)}{f\;\left(s_{1}\;|\;Y_{0}=1\right)P\;\left(Y_{0}=1\right)} \end{array}$$

Since (11) involves only counterfactuals observed on subjects with *Z*=1, *R*_1_ is statistically identifiable using subjects assigned to treatment *Z*=1. For example, *f* (*s*_1_ | *Y*_1_=1) can be estimated non-parametrically as the distribution of biomarker responses among treated subjects who experienced the outcome (*Y*=1⇒*Y*_1_=1), and *f* (*s*_1_) from biomarker responses among all treated subjects. *R*_0_,*RD*, and *RR* remain nonidentifiable because *S*_1_ is unobserved among subjects with *Z*=0 and *Y*_0_ is unobserved among those with *Z*=1, so that *f* (*s*_1_ | *Y*_0_=1) cannot be estimated from observed data. But even without further assumptions it is relatively straightforward to implement a sensitivity analysis which quantifies how the distribution of *S*_1_ | *Y*_0_=1 differs from the overall distribution of *S*_1_. An open-source web application for R that provides a graphical interface for sensitivity analysis under assumptions [SA1] and [SA2] is available at http://z.umn.edu/CESensApp.

[SA3] Monotonic treatment effect

Monotonicity assumptions restrict the joint distributions of counterfactuals by positing that they take on systematically lower (or higher) values under particular conditions. They are commonly applied in instrumental variable analyses and to study causal effects when there is a failure of compliance to treatment (see, e.g., Jin and Rubin [25]), where it is often assumed that compliance to treatment *Z*=1 is better when assigned to *Z*=1 than when assigned to treatment *Z*=0, and vice versa. Monotonicity assumptions can be applied to biomarkers, outcomes, and any other relevant variables that are measured after treatment has been assigned.

Individual-level monotonicity assumptions imply an ordering for two counterfactual random variables measured on the same subject, which places constraints on the joint distribution of counterfactuals and aids identifiability by ruling out certain combinations of outcomes. In a study comparing low-dose vitamin D supplementation (*Z*=1) to placebo (*Z*=0) for preventing occurrence of an episode of clinical depression (*Y*=1), it might be reasonable to assume that *P*(*Y*_*i*,1_≤*Y*_*i*,0_)=1 for all *i* since supplementation is very unlikely to result in a higher chance of clinical depression. Under this assumption, the counterfactual pair (*Y*_0_,*Y*_1_) is fully known for subjects with *Z*=1,*Y*=1 (1=*Y*_1_≤*Y*_0_=1) and *Z*=0,*Y*=0(0=*Y*_0_≥*Y*_1_=0), so that *P* (*Y*_1_=0 | *Y*_0_=0)=1 and *P* (*Y*_0_=1 | *Y*_1_=1)=1. While individual-level monotonicity assumptions involve the joint distribution of subject-specific counterfactual variables and are therefore untestable in general, they often have testable implications. In our example, the assumption *P* (*Y*_*i*,1_≤*Y*_*i*,0_)=1 implies that *P* (*Y*_1_=1)≤*P* (*Y*_0_=1), which is testable by considering the difference between *P* (*Y*=1 | *Z*=1) and *P* (*Y*=1 | *Z*=0).

Distribution-level monotonicity assumptions are weaker than individual-level assumptions, and give a stochastic ordering to counterfactual random variables. This ordering may relate the distributions two different counterfactuals (e.g., *S*_1_≥_*s*_*S*_0_), or two conditional distributions of the same counterfactual (e.g., *S*_1_ | *Y*_0_=0≥_*s*_*S*_1_ | *Y*_0_=1). For example, assuming that *S*_1_ | *Y*_1_=1≤_*s*_*S*_1_ | *Y*_0_=1 constrains the (nonidentifiable) CDF of *S*_1_ | *Y*_0_=1 to lie to the right of the (identifiable) CDF of *S*_1_ | *Y*_1_=1, thereby restricting the family of densities *f* (*s*_1_ | *Y*_0_=1) and potentially allowing (13) and (14) to be bounded using observed data.

In general, monotonicity is most likely to hold for intervention-placebo comparisons when the interventions (such as vaccines and educational/behavioral interventions) have few or no negative side effects. Monotonicity assumptions are less likely to be defensible for therapeutic agents that can be toxic or harmful (e.g., some types of chemotherapy) or when comparing two active treatments. Evaluating the validity of monotonicity assumptions can be tricky; for instance, in a re-analysis of data from the Lipid Research Clinics Coronary Primary Prevention Trial (LRC-CPPT) originally analyzed by Efron and Feldman [26], Goetghebeur and Molenberghs [27] observed that subjects with higher observed compliance to active treatment (cholestyramine, a cholesterol-lowering drug) were estimated to have worse response to lower doses than those with lower observed compliance, while the opposite was true for subjects with high compliance to placebo. They argued that this effect may have been due to the unpleasant gastrointestinal side effects of cholestyramine, which reduced compliance among those who had the least to gain by remaining compliant.

## Auxiliary data and augmented study designs

The fundamental challenge to the statistical identification of principal stratification estimands is the fact that joint counterfactual values are not observable. But in some situations it may be feasible to use auxiliary data, often in combination with modeling assumptions, to aid identifiability. Auxiliary data may arise from routine data collection, but they can also be obtained by modifying existing study designs.

### Using baseline predictors

The identifiability problem can be viewed as a missing data problem, where a subject receiving treatment *z* has *Y*_*z*_,*S*_*z*_, and $A_{z}^{\tau }$ observed but *Y*_1−*z*_,*S*_1−*z*_, and $A_{1-z}^{\tau }$ missing. From this perspective, the goal is to impute the missing counterfactual values.

One simple imputation method is to use an assumed regression model to “bridge” across treatments. Suppose that one can identify baseline covariate vectors *U* and *V* which correlate strongly with *S*_0_ and *S*_1_ and such that (*U*,*V*) can be measured on all subjects. Then one could consider regression models such as

(15) $$\begin{array}{l} g\;\left[E\;\left(S_{0}\;|\;U\right)\right]=\gamma_{0}+\gamma_{1}U\; \end{array}$$

(16) $$\begin{array}{l} g\;\left[E\;\left(S_{1}\;|\;V\right)\right]=\beta_{0}+\beta_{1}V\; \end{array}$$

Model (15) can be fit from subjects randomized to receive *Z*=0 (where *S*_0_ is identified) and be used to impute *S*_0_ values for subjects randomized to receive *Z*=1. Similarly, model (16) can be fit on those randomized to receive *Z*=1 (where *S*_1_ is identified) and produce imputed *S*_1_ values for subjects randomized to receive *Z*=0. This approach to imputation is valid since the Ignorable Treatment Assignments assumption guarantees that (*S*_0_,*S*_1_)⊥*Z* | *U*,*V*. The resulting joint (*S*_0_,*S*_1_) values can be used to fit an observed risk model such as

$$R_{z}\;\left(S_{0},S_{1}\right)=\alpha_{0}+\alpha_{1}z+\alpha_{2}\theta \;\left(S_{0},S_{1}\right)+\alpha_{3}z\times \theta \;\left(S_{0},S_{1}\right)$$

where *θ*(·) is some pre-defined function of *S*_0_ and *S*_1_.

As an example, Follmann [28] proposes an imputation strategy for HIV vaccine trials referred to as Baseline Irrelevant Vaccination (BIV). In that context, [SA1] and [SA2] are assumed to hold so that only *S*_1_ values need to be imputed. To produce a suitable *V* that strongly correlates with *S*_1_, Follmann suggests administering a rabies vaccine (the Baseline Irrelevant Vaccination) that does not affect the eventual vaccine-induced anti-HIV immune response, but serves as a proxy for each subject’s immune responsiveness. The resulting immune activation levels are used to fit a model such as (16). The BIV approach could also be adapted to cases where [SA2] does not hold, for instance an influenza vaccine trial where there is variability in the influenza-specific immune response due to previous exposure, and be incorporated into estimation methods such as that proposed in Zigler and Belin [20].

### Augmented study designs

Modified and novel study designs are another potential source of auxiliary data that can identify principal stratification estimands. The need to identify the joint values (*S*_0_,*S*_1_), (*Y*_0_,*Y*_1_), and so on for each individual leads naturally to the idea of crossover designs [29]. As an illustrative example, Donovan [30] evaluated the effect of the anticonvulsant Divalproex on Oppositional Defiant Disorder or Conduct Disorder in youth using a double-blind, placebo-controlled crossover trial. Suppose it were of interest to identify a surrogate endpoint (e.g., a score from a short mood questionnaire) for Divalproex’s ability to prevent episodes of explosive temper. In this setting, occurrence of the transient outcome is unlikely to interfere with future measurement of the biomarker of interest so that assumption [SA1] is satisfied. Furthermore, a suitable washout period between the Divalproex and placebo phases could minimize carryover effects on both the surrogate and the clinical endpoints. In this case, one might reasonably view the crossover data as if they were parallel realizations of (*S*_0_,*Y*_0_) and (*S*_1_,*Y*_1_) from the same subject, permitting full identification of *R*_1_, *R*_0_ and their contrasts.

**Figure 1:**
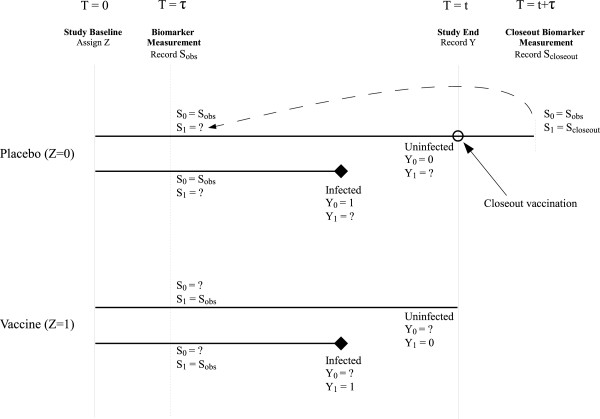
**Schematic of a vaccine trial design incorporating closeout placebo vaccination.** The four horizontal lines represent four subjects (two each assigned to placebo and active vaccine at time *T*=0). Subjects have biomarker *S*_*obs*_ measured at time *τ*, identifying *S*_0_ and *S*_1_ for placebo and vaccine recipients, respectively; the counterfactual biomarker values (*S*_1_ and *S*_0_ respectively) remain unidentified, as indicated by ‘?’. Subjects with the solid diamond represent those infected during the trial (yielding *Y*_0_=1 and *Y*_1_=1). Subjects assigned to placebo and uninfected during the trial (i.e., those with *Y*_0_=0) are eligible for closeout vaccination at the end of the study at time *T*=*t*. Post-closeout vaccination biomarker measurements *S*_*closeout*_ are obtained on these subjects at time *T*=*t*+*τ*. The dashed curved arrow represents the “time constancy” assumption that allows *S*_*closeout*_ to be used to identify *S*_1_, by definition the counterfactual measurement that would have been obtained at *T*=*τ* under assignment to the active vaccine. For ease of readability, this figure does not represent subjects who were infected prior to *τ* (i.e., those with *A*^*τ*^=0) and who would have *Y*=1 and *S* undefined.

For many clinical trials, it is impractical or unethical to use a simple crossover design. However, aspects of the crossover design can be used to augment standard parallel-arm designs to aid in the evaluation of surrogate endpoints. In addition to BIV, Follmann [28] also proposed a design modification known as Closeout Placebo Vaccination (CPV), wherein subjects assigned to receive the placebo at the beginning of the trial and who remain uninfected for the duration of the study are given the active vaccine (“closed out”) upon study completion and have their biomarker response measured. Wolfson and Gilbert [2] describe assumptions that permit this “closeout” value, say $S_{1}^{c}$, to be used in place of the unobserved *S*_1_ for these subjects. The key assumption is that of “time constancy”, i.e., that the underlying process generating observed *S* values has not changed over the course of the study among subjects who received the placebo. This assumption may be reasonable in trials where the biomarker of interest measures an aspect of a biological system which remains relatively stable over time in the trial population (e.g., the immune system among adults aged 20 - 50 in an HIV vaccine trial). Figure 1 provides an overview of the Closeout Placebo Vaccination design.

Data from a Closeout Placebo Vaccination design allow the identification and estimation of the distribution of *S*_1_ | *Y*_0_=0, since uninfected placebos are precisely those with *Y*_0_=0. Using the relation

(17) $$f\;\left(s_{1}\;|\;Y_{0}=1\right)=\frac{f\;\left(s_{1}\right)-f\;\left(s_{1}\;|\;Y_{0}=0\right)P\;\left(Y_{0}=0\right)}{P\;\left(Y_{0}=1\right)}$$

it is also possible to identify *f* (*s*_1_ | *Y*_0_=1) and hence, if assumptions [SA1] and [SA2] hold, (11)-(14) can be fully identified using these augmented data.

Information from both baseline covariates and closeout vaccination can be combined to improve identifiability and efficiency. Huang et al. [21] present a novel pseudo-score approach to estimation of *R*_1_ and *R*_0_ in the context of HIV vaccine trials, and compare the efficiency of using only baseline covariates with using baseline covariates plus data from Closeout Placebo Vaccination. The approach accommodates a two-phase sampling strategy in which a subset of vaccinated subjects and uninfected placebo recipients are selected to have tissue samples assayed to obtain information on *S*.

Closeout designs need not be limited to the context of vaccine trials; they may be of use in any placebo-controlled trial where the “time constancy” assumption is reasonable. These designs are most feasible when either a) blinding can be maintained during the closeout period, or b) the biomarker of interest is a physiological parameter (e.g., elimination rate of a particular compound) which is unlikely to be strongly affected if participants are unblinded to treatment status.

## Example scenarios

Thus far, we have described key assumptions and sources of auxiliary data that help identify principal stratification estimands and thereby facilitate assessment of the surrogate value of a biomarker. In this section, we present four hypothetical scenarios where one might wish to assess surrogate value. We discuss which of the above assumptions and augmented designs are plausible or feasible in each scenario, and show that assessing surrogate value via principal stratification in the four scenarios is straightforward, moderately difficult, somewhat challenging, and extremely challenging, respectively. Table 1 summarizes the results of this section.

**Table 1:** Difficulty of evaluating surrogate endpoints in four scenarios

**Scenario**	**Endpoint** ***Y***	**Possible surrogate** ***S***	**[SA1]**	**[SA2]**	**[SA3]**	**BP**	**Closeout**	**Difficulty**
HIV vaccine trial	Infection with HIV	Immune response	∼	✓	✓	∼	✓	Low/Moderate
Influenza vaccine trial	Infection with influenza	Immune response	✓		✓	✓	∼	Moderate
Surgery for CHF	Survival	Admission-free survival	✓			∼		Moderate/High
Treatment for CVD	Survival	Blood biomarkers				∼	∼	High

✓  indicates assumptions ([SA1]-[SA3]) and auxiliary data collection strategies (BP = use of baseline predictors, Closeout = closeout design) which will typically be plausible in each scenario. ∼ indicates assumptions and strategies which may be plausible in certain special cases which are described in the text.

**[ Scenario 1 - HIV vaccine trial ]***Clinical endpoint Y:* Infection with HIV*Proposed surrogate S:* HIV-specific immune response For HIV vaccine trials, the surrogate endpoint problem consists of identifying specific immune response profiles that quantify the degree to which a subject is protected against HIV infection after receiving the vaccine. As detailed in several sections of this paper, HIV vaccine trials possess characteristics that simplify the assessment of surrogate value and may even in some cases allow full statistical identification of principal stratification-based estimands.

*Assumptions assessment:***[SA1]**: Many previous HIV vaccines required subjects to undergo a sequence of injections, and hence peak immunity was not established until several months after the start of the trial. Since the immune response to the vaccine cannot be measured in the presence of an active HIV infection, [SA1] may be questionable. Future formulations may require fewer injections and induce relevant immune responses more quickly, so that [SA1] may be valid.

**[SA2]**: As described in the section introducing [SA2], healthy volunteers who receive a placebo vaccine have no HIV-specific immune cells and hence it is reasonable to assume that *S*_0_=*c*, so [SA2] holds.

**[SA3]**: Since vaccines are designed for prevention of disease in the general population, tolerance for vaccine side effects is low and it may be plausible to assume a monotonic vaccine treatment effect, e.g., *P*(*Y*_1_≤*Y*_0_)=1. However, it is worth noting that some early vaccine trials showed weak evidence of an “enhancement” effect where vaccinated subjects were in fact more likely to be infected than placebo recipients. While this would negate [SA3] and make surrogate assessment more challenging, in practice it is unlikely that there would be great interest in understanding the relevant surrogates for such a vaccine.

*Auxiliary data and augmented designs:* As detailed above, the Baseline Irrelevant Vaccination and Closeout Placebo Vaccination designs were proposed first in the context of HIV vaccine trials, and so may provide useful tools for identifying principal stratification estimands.

**[ Scenario 2 - Influenza vaccine trial ]***Clinical endpoint Y:* Flu infection in a given season*Proposed surrogate S:* Immune response to vaccine

Rapid prototyping of influenza vaccines relies on the identification of reliable immune biomarkers which reflect the degree of protection offered by the vaccine. Trials of influenza vaccines share many characteristics with HIV vaccine trials, with the chief exception being that subjects enrolled in these trials are likely to have been previously infected with influenza.

Assumptions assessment:

**[SA1]**: Participants in influenza vaccine trials may become infected with the flu before their immune response to the vaccine is measured. However, the relatively short time frame between influenza vaccination and peak immune response (reported as 4 - 9 days [31]) may limit the degree to which this assumption will be violated.

**[SA2]**: Most subjects enrolled in influenza vaccine trials will have been infected previously with influenza, and so there is likely to be variability in *S*_0_ due to different levels of immune cross-reactivity with the strain of interest. [SA2] is therefore unlikely to be plausible.

**[SA3]**: Influenza vaccines are unlikely to cause harm, and are generally not believed to increase susceptibility to influenza infection. However, some caution is warranted before blindly adopting the monotonicity assumption [SA3]; for instance, a trial participant who remains infection-free after multiple exposures to influenza-infected individuals may conclude that he or she received the active vaccine and hence take fewer precautions to prevent future exposure, increasing his or her likelihood of infection.

*Auxiliary data and augmented designs:* Closeout placebo vaccination may be possible in influenza vaccine trials, though its value may be limited if a substantial fraction of study subejcts acquire influenza during the study and hence are ineligible to be “closed out”. A modification of the baseline predictor strategy also is possible [28], since the pre-vaccination levels of the biomarker of interest may be very highly correlated with *S*_0_, and can be measured on all subjects.

[ Scenario 3 - Randomized trial of surgical treatments for patients with congestive heart failure ]

*Clinical endpoint Y:* 3-year overall survival

*Proposed surrogate S:* 1-year admission-free survival

Congestive heart failure is the leading cause of hospitalization in people over the age of 65 [32]. There is substantial debate on the best course of management for these patients, particularly those whose symptoms are relatively mild. One option is surgery (coronary artery bypass or valve reconstruction), though these operations carry non-trivial risk and may not improve long-term outcomes. In a hypothetical trial evaluating the benefits of immediate surgery versus, for example, watchful waiting, it may be of interest to assess whether early outcomes are indicative of a survival benefit after three years. In this case, a candidate “biomarker” could be the rate of admission-free survival at one year, i.e., the proportion of subjects who are still alive and have not been admitted to a hospital due to heart failure symptoms.

Assumptions assessment:

**[SA1]**: Use of an earlier-occurring version of the clinical endpoint of interest as a potential surrogate, rather than a lab-measured biomarker, can simplify the assessment of surrogate value. In this scenario, by definition the clinical endpoint cannot occur before the candidate surrogate is measured, and hence [SA1] holds.

**[SA2]**: Clearly, one would expect variability in 1-year admission-free survival in both study arms, hence [SA2] is implausible.

**[SA3]**: Surgery in patients with congestive heart failure can be risky, and the amount of morbidity and mortality associated with immediate surgery could conceivably outweigh the improvement in symptoms experienced by those whose surgeries are successful. Hence the monotonicity assumption is unlikely to hold for either 1-year admission-free survival or 3-year overall survival.

*Auxiliary data and augmented designs:* The major demographic factors associated with admission and survival rates for heart failure have been studied extensively, so it may be feasible to use these factors to construct a model to impute *S*_0_ and *S*_1_ among subjects randomized to treatments *Z*=1 and *Z*=0 respectively.

Due to the temporal nature and ordering of the proposed surrogate and clinical outcome, crossover and closeout designs are not possible in this setting.

[ Scenario 4 - Cardiovascular drug therapy ]

*Clinical endpoint Y:* Occurrence of cardiovascular events

*Proposed surrogate S:* Various blood-based biomarkers

For many years there has been much interest in identifying biomarkers of cardiovascular disease [33,34]. Assessing whether these biomarkers are valid surrogates for the clinical effects of cardiovascular disease medications can be challenging. Among many difficulties, the biomarkers themselves might not be well understood until years after their discovery; such was the case for soluble thrombomodulin 2 (ST2) [33] and C-reactive protein (CRP) [35], and may be the case for many currently proposed biomarkers (see, e.g., Table eight in Vasan [34]). Further, the mechanisms of action of many relatively successful cardiovascular medications have not yet been described fully.

Assumptions assessment:

**[SA1]**: Treatments for cardiovascular disease may not achieve their full effects on biomarkers for weeks or months, during which time cardiovascular events may occur and thereby preclude the measurement of the markers of interest. Violations of [SA1] are particularly likely in studies of populations where the rate of severe cardiovascular disease and the incidence of cardiovascular events is high.

**[SA2]**: Many cardiovascular biomarkers of interest are nonspecific, reflecting changes in a number of biological processes. For example, CRP is a generalized marker of inflammation, and hence may be elevated due to transient conditions such as a bacterial or viral infection or noncardiovascular chronic conditions such as cancer malignancy. [SA2] is therefore unlikely to be satisfied.

**[SA3]**: Treatment of cardiovascular disease has progressed to the point where several effective treatments exist and new drugs are evaluated against standard-of-care regimens. Therefore, in many cases it may be unreasonable to assume that treatment effects on either the biomarker of interest or the clinical endpoint are monotonic in favor of the new drug. But when comparing standard-of-care and an “augmented” standard-of-care including a new medication, [SA3] may be warranted provided there are few concerns about the medications involved producing a harmful interaction.

*Auxiliary data and augmented designs:* When two different active treatments are being compared, closeout designs may be difficult to justify because the biomarker levels achieved by an individual that was randomized to treatment *Z*=0 and was subsequently “closed out” with treatment *Z*=1 may not reflect the levels that would have been achieved had that individual been randomized to treatment *Z*=1 initially. However, a closeout design analogous to Closeout Placebo Vaccination may be feasible when treatment *Z*=0 is standard-of-care and *Z*=1 is standard-of-care plus a new medication. In all cases, the “time constancy” assumptions that allow biomarkers measured at the end of the study to substitute for biomarkers measured shortly after randomization must be evaluated carefully, since the physiological systems influencing biomarkers may undergo rapid changes with aging and as cardiovascular disease progresses. The baseline predictor approach requires fewer assumptions and trial design modifications, but its utility may be limited since the predictors of most cardiovascular biomarkers are often poorly understood.

## Conclusion

The principal stratification approach to evaluating surrogate endpoints relies on estimands that capture causal effects of interest but may not be statistically identifiable. Exploring the identifiability of these estimands under a variety of assumptions reveals that the nature of the data-generating process and the constraints imposed by randomized trial designs have a major impact on the ability to use statistical modeling to assess the value of surrogate endpoints. When many of the assumptions outlined above are plausible or auxiliary data are available, principal stratification estimands may be identifiable or nearly identifiable such that a straightforward sensitivity analysis is possible. In such settings, statistical analysis of data arising from a well-designed Phase III trial (possibly incorporating one of the aforementioned enhanced study designs) may provide insights into surrogacy. Conversely, when biomarkers are not well understood, when the potential side effects of the proposed treatment are poorly characterized, or when treatment effects on biomarkers are only fully achieved a long time after randomization, it may not be possible to identify the relevant risk estimands without several strong and untestable assumptions. In these more difficult cases, statistical analyses will be of limited use in the evaluation of candidate surrogates, and researchers must rely more heavily on findings from laboratory and clinical science.

Though one might be tempted to conclude from this paper that the search for reliable surrogate endpoints is doomed to failure in many areas of biomedical research, we do not subscribe to such a pessimistic view. Rather, we believe that increased awareness of how the characteristics of diseases, treatments, and study logistics combine to affect the ability to identify surrogate endpoints can assist with the planning, implementation, and analysis of major trials. By incorporating novel design concepts and by carefully assessing the validity of key assumptions, we believe that future studies will be able to coax surrogate needles out of the ever-growing biomarker haystack.

## Competing interests

The authors declare that they have no competing interests.

## Authors’ contributions

JW drafted the manuscript based on research performed in collaboration with LH. Both authors read and approved the final manuscript.
